# Fault-scale crustal structure across the Dunhua-Mishan fault (Tanlu northern segment) constrained from teleseismic P-wave receiver functions

**DOI:** 10.1038/s41598-024-56620-2

**Published:** 2024-03-09

**Authors:** Qian Liu, Ziqiang Lü, Guangwei Zhang, Mingwen Lu

**Affiliations:** 1 College of Mining, Liaoning Technical University, Fuxin, China; 2 Key Laboratory of Crustal Dynamics, National Institute of Natural Hazards, Ministry of Emergency Management of China, Beijing, China

**Keywords:** Geophysics, Seismology

## Abstract

The Dunhua-Mishan fault, located in the northern segment of the Tanlu fault zone, experienced multiple tectonic processes associated with the effects of the Pacific Plate subduction and the Indo-Asia collision. The high-resolution fault-scale structure is critical for understanding the fault evolution and potential fault damage. However, the well-defined deep structure of the Dunhua-Mishan fault is still unclear due to the lack of the dense seismic array. In this study, we construct a high-resolution P-wave receiver function imaging based on linear dense seismic array across the fault. Our results reveal the strong Moho depth variation across the Dunhua-Mishan fault zone. The slightly higher Vp/Vs ratio values within the fault zone indicate the presence of a small amount of mafic crust composition. Interestingly, the significant double positive Ps converted phases are observed within the fault zone, which may represent double Moho discontinuities. The double Moho structure may be related to multiple significant tectonic activities in the Tanlu northern segment. These newly observed structures provide new seismic constraints on the formation and evolution of the Tanlu fault zone and probably reflect that the lithospheric structure of the Dunhua-Mishan fault has been modified by a series of tectonic processes.

## Introduction

**Figure 1:**
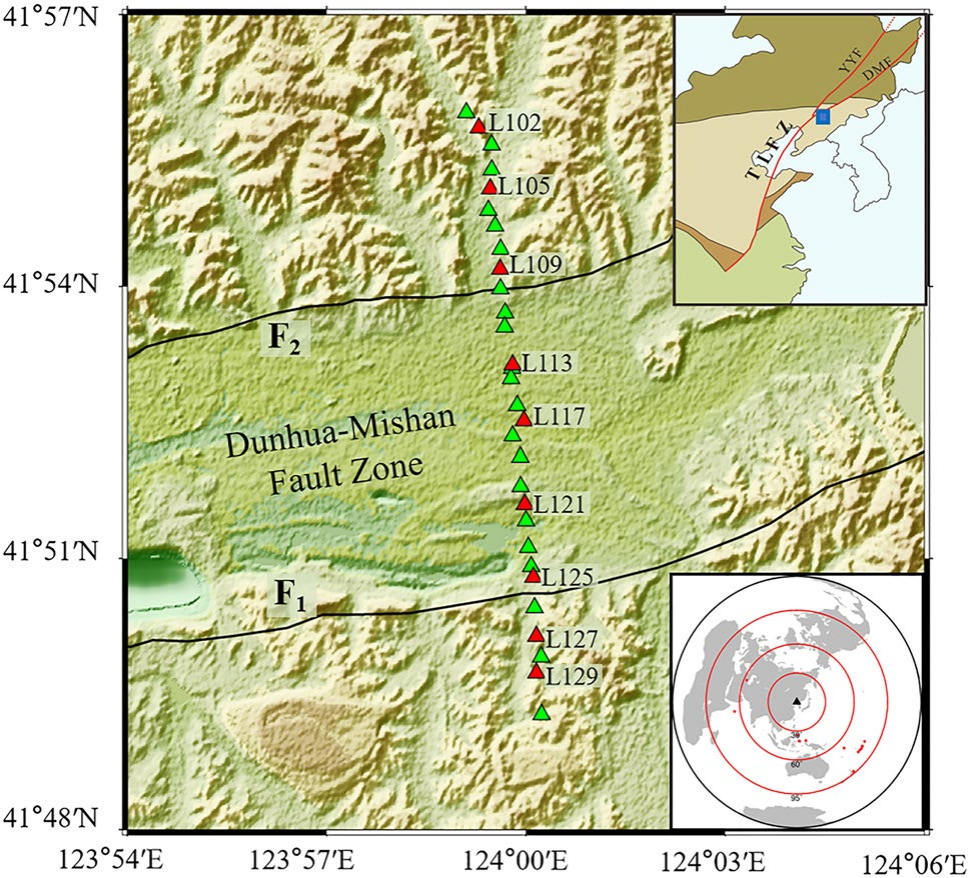
The linear seismic array (green and red triangles) and faults (black lines) in the study region. F1, the south branch of the Dunhua-Mishan fault. F2, the north branch of the Dunhua-Mishan fault. The blue square in the top right corner inset displays the location of the study region. The northern segment of the Tanlu fault zone (TLFZ) is composed of the Yilan-Yitong Fault (YYF) to the west and the Dunhua-Mishan Fault (DMF) to the east. The inset in the bottom right corner represents the location of teleseismic events (red dots) used for the calculation of receiver functions within 30° and 95° epicentral distance. The black triangle marks the center of the study region. Imagery is available from the U.S. Geological Survey (https://lpdaac.usgs.gov/products/srtmgl1v003). Figure made with Generic Mapping Tools^8^ (GMT v.6.4.0: https://www.generic-mapping-tools.org).

Extending over a distance of ~ 2400 km, the Tanlu fault zone is the largest NNE-SSW trending fault stretching from the Dabie orogenic belt to the Sino-Russian border^1,2^. In general, the Tanlu fault zone can be classified into northern, central, and southern segments based on distinct tectonic characteristics and evolutional history^2,3^. The northern segment of the Tanlu fault zone in northeast China is mainly composed of the Yilan-Yitong Fault to the west and the Dunhua-Mishan Fault to the east^4^ (Fig. 1). As a significant branch fault, the Dunhua-Mishan fault is a left-lateral strike-slip fault extending about 1000 km in northeast China and undergone a series of complex and multistage tectonic events with a series of microseismic activity^5–7^(Fig. S1). It is a specific branch offering valuable insights into the geological tectonic evolution of the northern segment of the Tanlu fault zone.

The northern segment of the Tanlu fault zone experienced multiple tectonic episodes driven by the subduction movement of the ancient Pacific plate to the Eurasian plate and the Indo-Asian continental collision^1,9–14^. During the early Cretaceous period, large-scale sinistral strike-slip movements occurred along the northern segment of the Tanlu fault zone, resulting in a total offset distance was approximately 150 km^1,15,16^. From the Cretaceous to the Neogene, the Tanlu northern segment underwent a series of episodes of compression and extension, as a consequence, numerous graben basins formed followed by subsequent uplift processes with intense magmatic events and widespread lithospheric expansion^17,18^. In the late Quaternary, the northern segment of the Tanlu fault zone experienced further tectonic activity, leading to the current geological structure configuration^7,19^.

Previous studies investigated the crustal and upper mantle structures to better understand the lithosphere formation and modification across the northern segment of the Tanlu fault zone. The seismic azimuth anisotropy showed that the fast directions within the northern segment of the Tanlu fault zone were systematically shifted to NW–SE, which may reflect the extension of the lithosphere since the late Mesozoic^20–23^. A deep seismic reflection profile in the northern region of the Dunhua-Mishan fault within the Jiamusi-Mudanjiang block revealed that the Dunhua-Mishan Fault penetrate the crust into the upper mantle^24^. Based on a 2-D viscoelastic model, a large-scale post-seismic deformation following the 2011 Mw 9.0 Tohoku–Oki earthquake was observed across the northern segment of the Tanlu fault zone, which exists disparity between the two sides of Tanlu northern segment due to heterogeneity in viscosity structures^25^. The geochemical study revealed that the deep section of the northern segment of the Tanlu fault zone showed obvious upward migration of H_2_, Hg, and Rn gases, which is influenced by regional tectonic stress field, reflecting the fault has a certain level of activity^26^. Additionally, integrated 1254 seismic events with M_L_ ≥ 2.0, 191 mechanism solutions from January 1964 to January 2008 in the northern segment of Tanlu Fault zone and surrounding regions study revealed the relatively active seismicity along Tanlu northern segment^27,28^, corresponding to the presence of a low-velocity and high-conductivity layer in the crust-upper mantle^29,30^.

Region-scale receiver function results revealed the widespread crustal thinning and relatively complex Poisson’s ratio variation in the northern segment of the Tanlu fault zone, which suggests the crust may undergo a strong stretching and thinning process^31,32^. Moreover, the common conversion point (CCP) imaging revealed Moho migration and offset beneath the northern segment of the Tanlu fault zone^33–35^, which indicates the Tanlu northern segment extends to the uppermost mantle, providing a channel for the upwelling of magmatic materials. However, these results mainly focus on large-scale features with relatively low lateral resolution due to the limited distribution of seismic stations.

The detailed fault zone structure is essential for comprehending fault evolution and assessing potential hazards. In this study, with the significant improvement of seismic network coverage along the northern segment of the Tanlu fault zone, we construct the deep crustal structure using the receiver function imaging based on a dense linear seismic array across the Dunhua-Mishan fault. Our results demonstrate the lateral distribution of the Moho depth and the crustal Vp/Vs ratio linked to the different regional tectonic segments. Particularly, we observe the double Moho structure beneath the fault zone, which may be related to the historical tectonic processes of the Tanlu northern segment. These new findings provide more structural constraints on the formation and evolution of the northern segment of the Tanlu fault zone.

## Results

**Figure 2:**
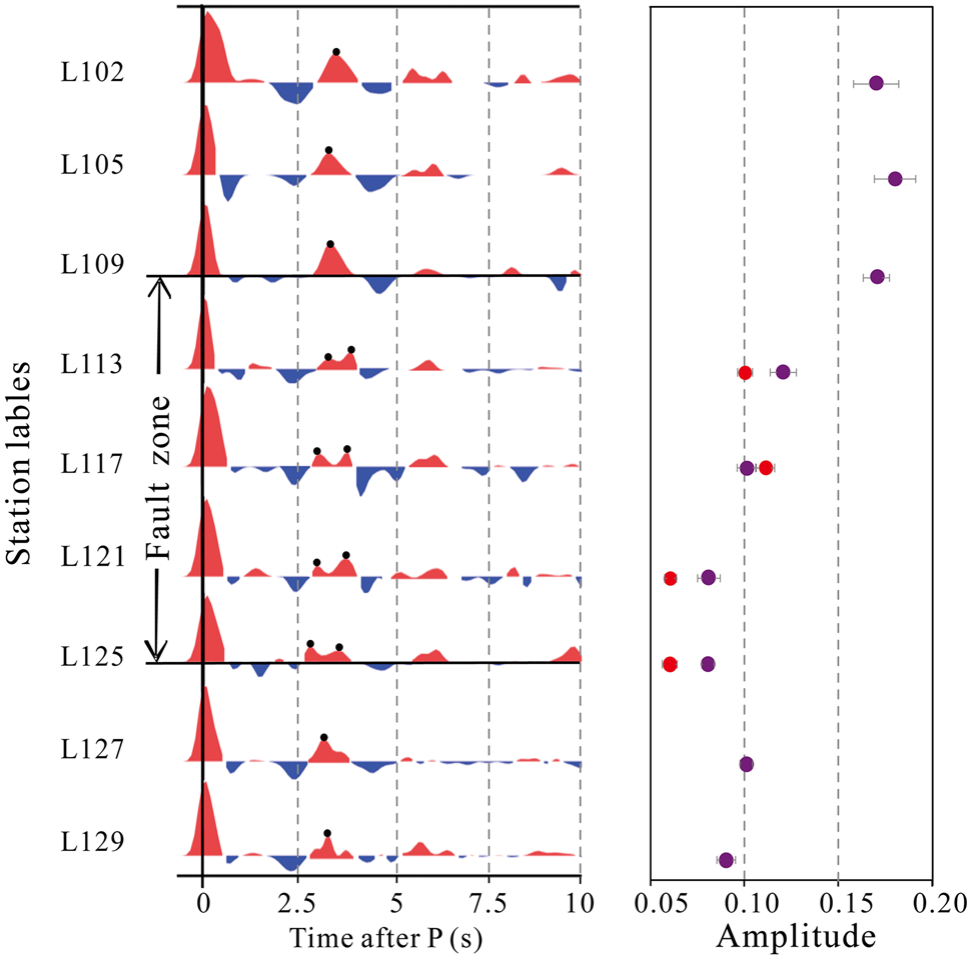
Examples of observed stacked radial component receiver functions at each station. The black dots represent Ps arrival time in stacked radial component receiver functions. The purple and red dots indicate the amplitude of lower Moho and upper Moho, respectively. The error bars represent the uncertainty of Ps phase amplitudes. See station locations in Fig. 1.

**Figure 3:**
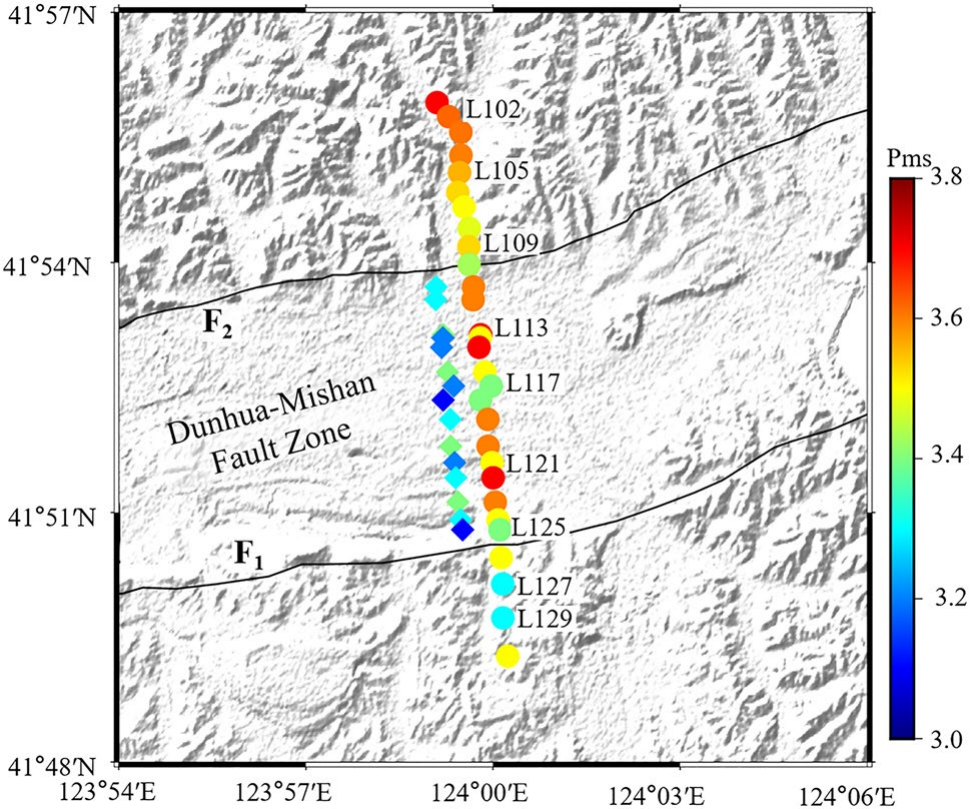
The identification time of the Moho Ps converted seismic phase. The rhombuses represent the first identification time of the Ps converted seismic phase and the circles represent the second identification time of the Ps converted seismic phase within the fault zone. See the description in Fig. 1 for other symbols. Imagery is available from the U.S. Geological Survey (https://lpdaac.usgs.gov/products/srtmgl1v003). Figure made with Generic Mapping Tools^8^ (GMT v.6.4.0: https://www.generic-mapping-tools.org).

The receiver function results illustrate complex fault-scale crustal structure variations across the Dunhua-Mishan fault in the Tanlu northern segment benefiting from the integration of the dense seismic array. Figure 2 shows the example of individual stacked receiver functions and uncertainties along the linear seismic array. We observe the significant double positive Ps converted phases within the fault zone, in contrast, the simple Ps converted phases are discovered in the surrounding mountain regions. The corresponding Ps uncertainty at each station varies within a small range of 0.016–0.03 s (Fig. 2). We hand-picked the double Ps converted phase arrivals within a time window of ~ 3.1–3.7 s (Fig. 3). The earlier Ps converted phase arrivals are observed at ~ 3.1 s, and the following Ps converted phases are shown at ~ 3.7 s in the fault zone. The average arrival difference between the double Ps converted phases is ~ 0.3 s.

**Figure 4:**
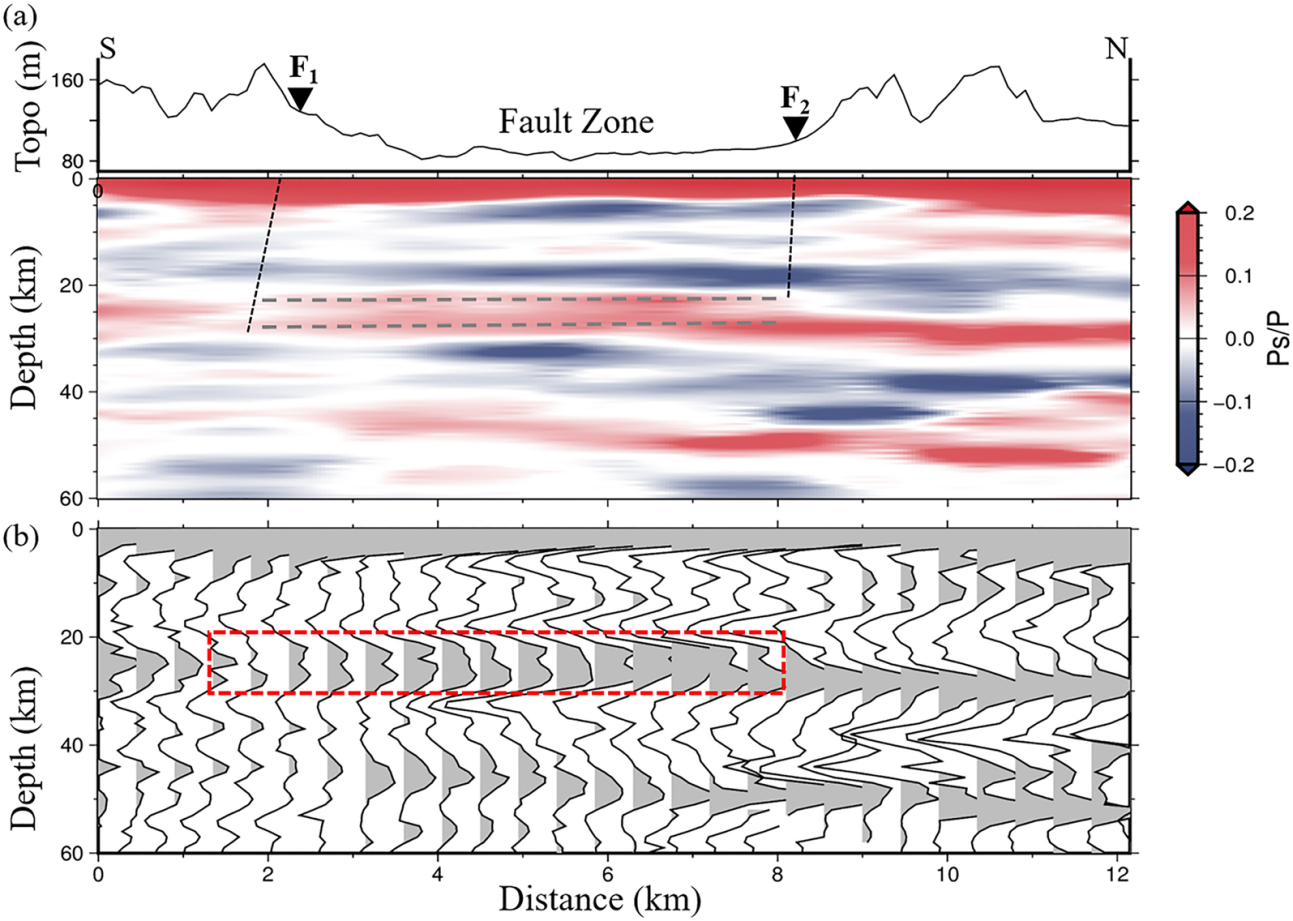
(**a**) The CCP image of the crustal structure in the study region. Red and blue colors show positive and negative amplitudes, respectively. The top black curve is topography. F_1_, the south branch of the Dunhua-Mishan fault. F_2_, the north branch of the Dunhua-Mishan fault. Two gray dashed lines represent the double Moho. (**b**) The CCP image showing in the stacked traces. Red dashed frame marks the double Moho within the fault zone.

The linear dense seismic array is nearly perpendicular to the fault, which can clearly demonstrate the Moho depth variations in different tectonic segments. Detailed crustal structure variation performed CCP imaging with different sliding distance along the profile are described in Fig. S2. The CCP imaging shows the lateral variation of Moho discontinuities at depths of 26–31 km with low uncertainties, and the Moho depth pattern correlates well with major tectonic segments across the profile (Figs. 4 and S3). The relatively simple and continuously traced Moho Ps conversions can be observed beneath the mountain ranges, especially in the north of the fault, whereas the double Ps conversions at Moho depth were imaged within the fault zone (Fig. 4b). In addition, it seems to be a slight offset in the south of Dunhua-Mishan fault. In order to improve the reliability of our results, we also consider the 3D velocity model for CCP stacking^36^, which is generally consistent with the current IASP91 model (Fig. S4).

**Figure 5:**
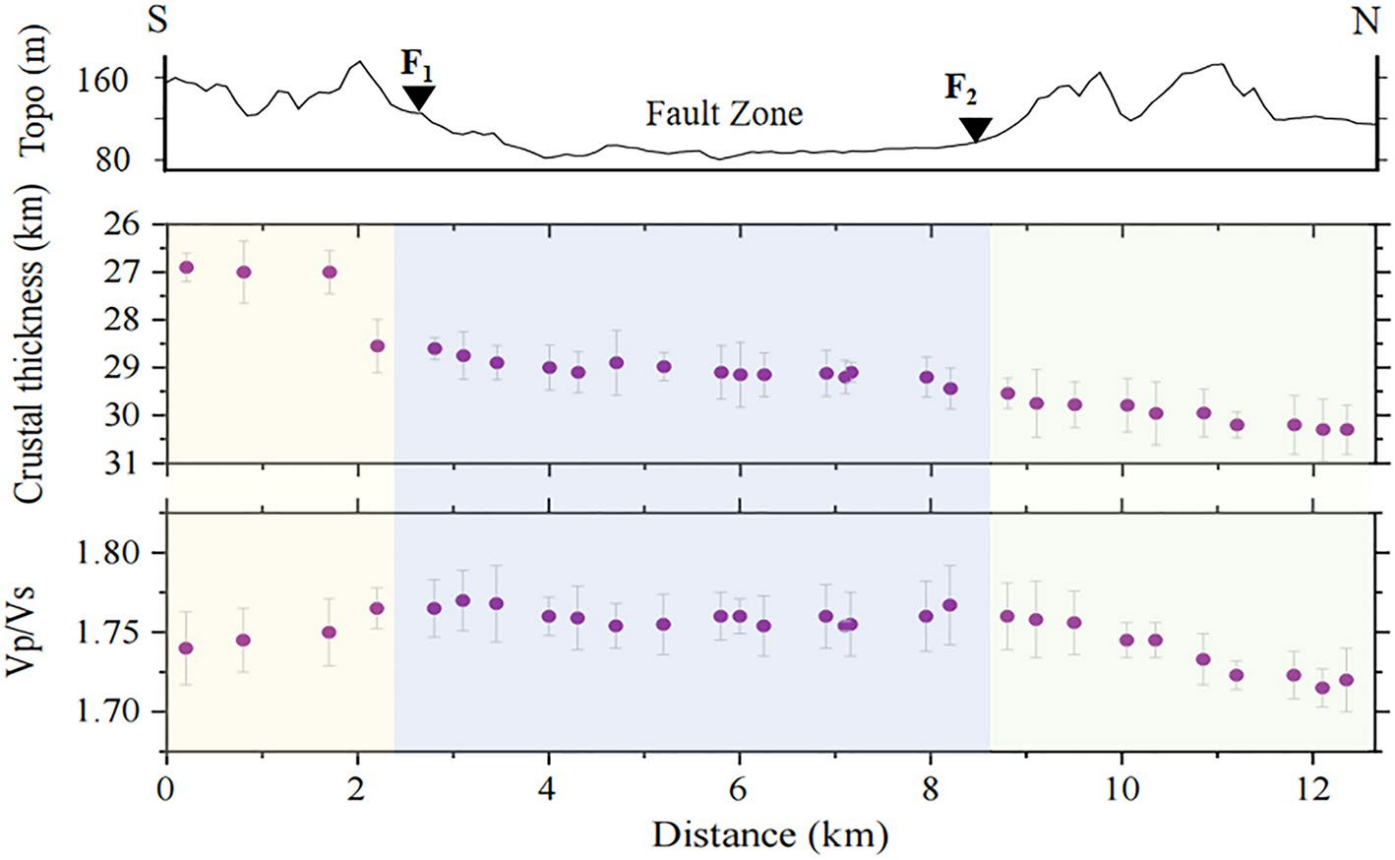
Crustal thickness and Vp/Vs ratio in the study region derived from H-κ stacking. The purple dots indicate the crustal thickness value and Vp/Vs ratio for each station, respectively. The gray short lines represent the corresponding errors. See the description in Fig. 1 for other symbols.

**Figure 6:**
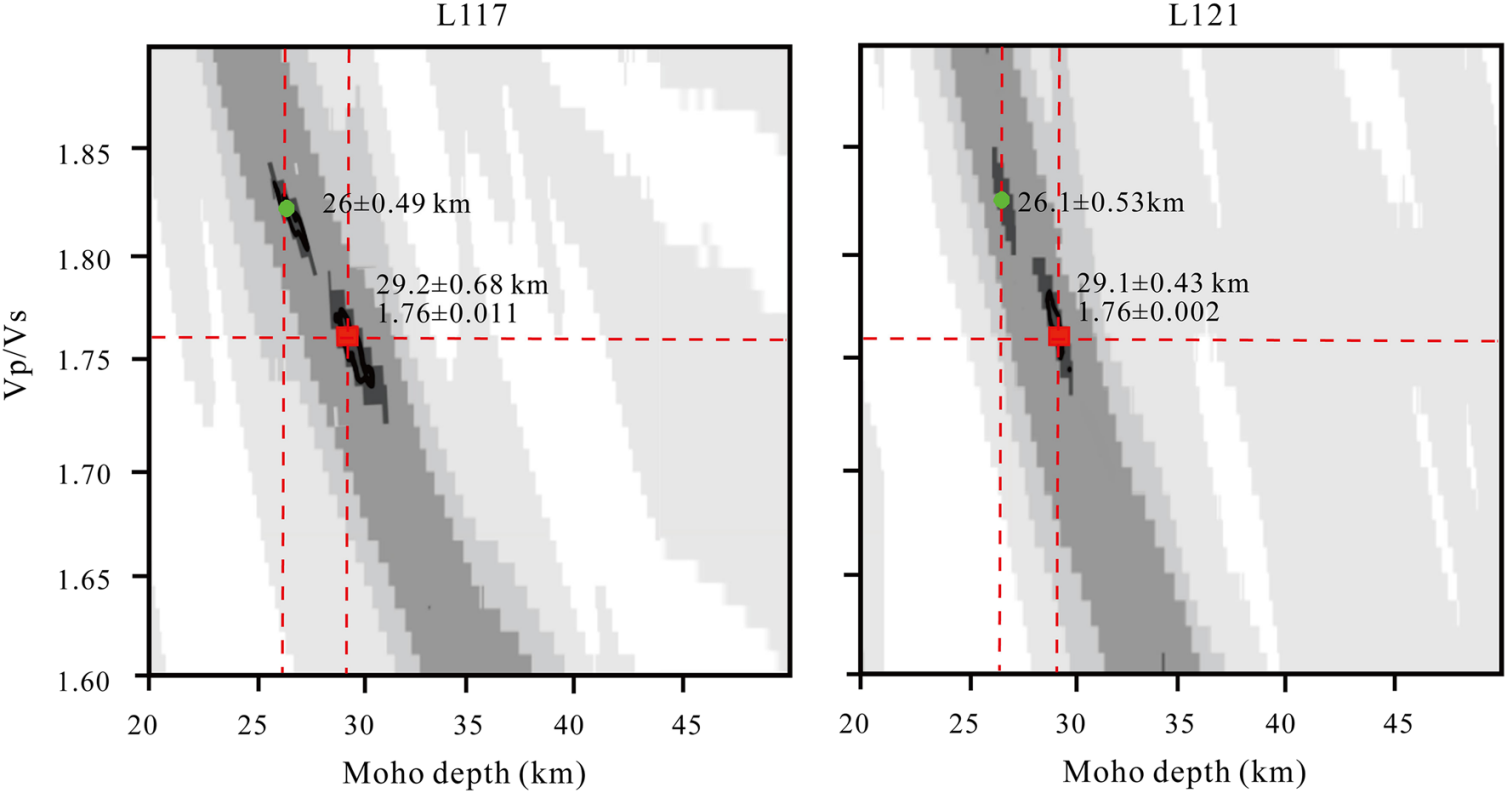
Results of the H-κ stacking at L117 and L121 stations within the fault zone. The small green dots represent the upper Moho depths. The small red squares represent the lower Moho depths and corresponding Vp/Vs ratio.

The crustal thickness and Vp/Vs ratio along the linear seismic array were obtained by performing the H-κ stacking algorithm (Figs. 5 and S5). In the southern mountain region, the crust thickness changes significantly, increasing from 26.9 to 28.6 km, and the Vp/Vs ratio also increases from 1.74 to 1.77 (Fig. 5). The average crustal thickness of the fault zone is approximately 29 km, the Vp/Vs ratio is higher compared to the surrounding regions. In the northern mountain region, the deepening Moho is observed with average crust thicknesses of about 30.1 km, and the Vp/Vs ratio decreases from 1.76 to 1.71 along the profile. We can also observe distinct double Moho at L117 and L121 stations within the fault zone (Fig. 6). These results are consistent with the CCP imaging, indicating that the observed double Moho are robust due to the linear dense seismic array.

## Discussion

The P-wave receiver functions are widely used in constraining the velocity discontinuities, especially the Moho depth distribution, which provides important information on the crustal deformation and dynamical processes^37,38^. Combined with the newly deployed linear dense seismic array, we can observe the fault-scale crustal structure beneath the Dunhua-Mishan fault zone. Specifically, the observed double Ps converted phases within the fault zone may provide additional constraints on the evolution of the Tanlu northern segment.

Regional-scale receiver functions reveal the average Moho depth is ~ 29 km in this study region^31–33,39^, however, it is difficult to reveal the detailed characteristics of the fault-scale crustal structure due to the sparse station coverage. Our results demonstrate a strong correlation between the Moho depth variations and regional tectonic segments across the fault. Beneath the southern and northern mountain regions, the Moho variation is relatively flat and single in comparison with the complex Moho depth patterns within the fault zone, indicating the fault experienced different degrees of crustal modification.

We observe a weak and seemingly discontinuous Moho pattern between the fault zone and the south branch of the Dunhua-Mishan fault (F_1_). It is noted that the previous regional shear-wave velocity model and deep seismic reflection could not reveal this small-scale feature due to the low resolution^23,34^. The observed weak Moho discontinuity beneath the south branch of the Dunhua-Mishan fault indicates fault may be a lithospheric scale fault. The previous magnetotelluric study revealed sub-vertical low-resistivity zones penetrating the entire lithosphere beneath the Dunhua-Mishan fault^40^, which also supports the Dunhua-Mishan fault extends to the uppermost mantle. The lithospheric scale fault could be a channel for material and energy exchanges along the fault.

The crustal Vp/Vs ratio, a crucial parameter for determining crustal structure and composition^41^, is predominantly influenced by rock physical properties and composition^42^. Generally, the large Vp/Vs ratio indicates that the crustal composition is mainly mafic rocks, whereas the small Vp/Vs ratio indicates that the crust is composed mainly of felsic rocks. Fluid or local melting significantly decreases shear wave velocity more than compressional wave velocity, leading to an increase in the Vp/Vs ratio.

The H-κ stacking results reveal a relatively higher Vp/Vs ratio (~ 1.76) within the Dunhua-Mishan fault zone compared with surrounding mountain regions. These results are slightly higher than the average Vp/Vs ratio (1.75) of typically continental crust^41,42^ to some extent, indicating the presence of a mafic crust. The existence of large-scale basalt along the Dunhua Mishan fault, as confirmed by geochemical studies^43,44^, further supports our observations. The mafic mantle basaltic magma intrudes into the crust along the weak fault zone, which contributes to the observed higher Vp/Vs ratio within the fault zone, highlighting the fault as a lithospheric channel for thermal upwelling^45^.

More importantly, we observe a double Moho structure within the Dunhua-Mishan fault zone, providing significant evidence for the evolution of the fault. The double Moho provides important insights into the internal structure of the fault, with its genesis tied to a series of complex geodynamic processes. Although observed double Moho within the Dunhua-Mishan fault zone is located in the sediments with low elevation, and the total sediment thickness is ~ 1.8 km^46^. Based on the synthetic test, the corresponding multiple phases are not observably affect the identification of the double Moho (Fig. S6). Generally, the explanations for the origins of the double Moho in the continent primarily attribute to the following aspects: (1) The continental collision forms Moho doublet^47^. (2) Long-term accumulation of the sustained compression leads to the multiple Moho structure^48^. (3) Intrusion of magmatic material results in the formation of the double Moho^49^.

**Figure 7:**
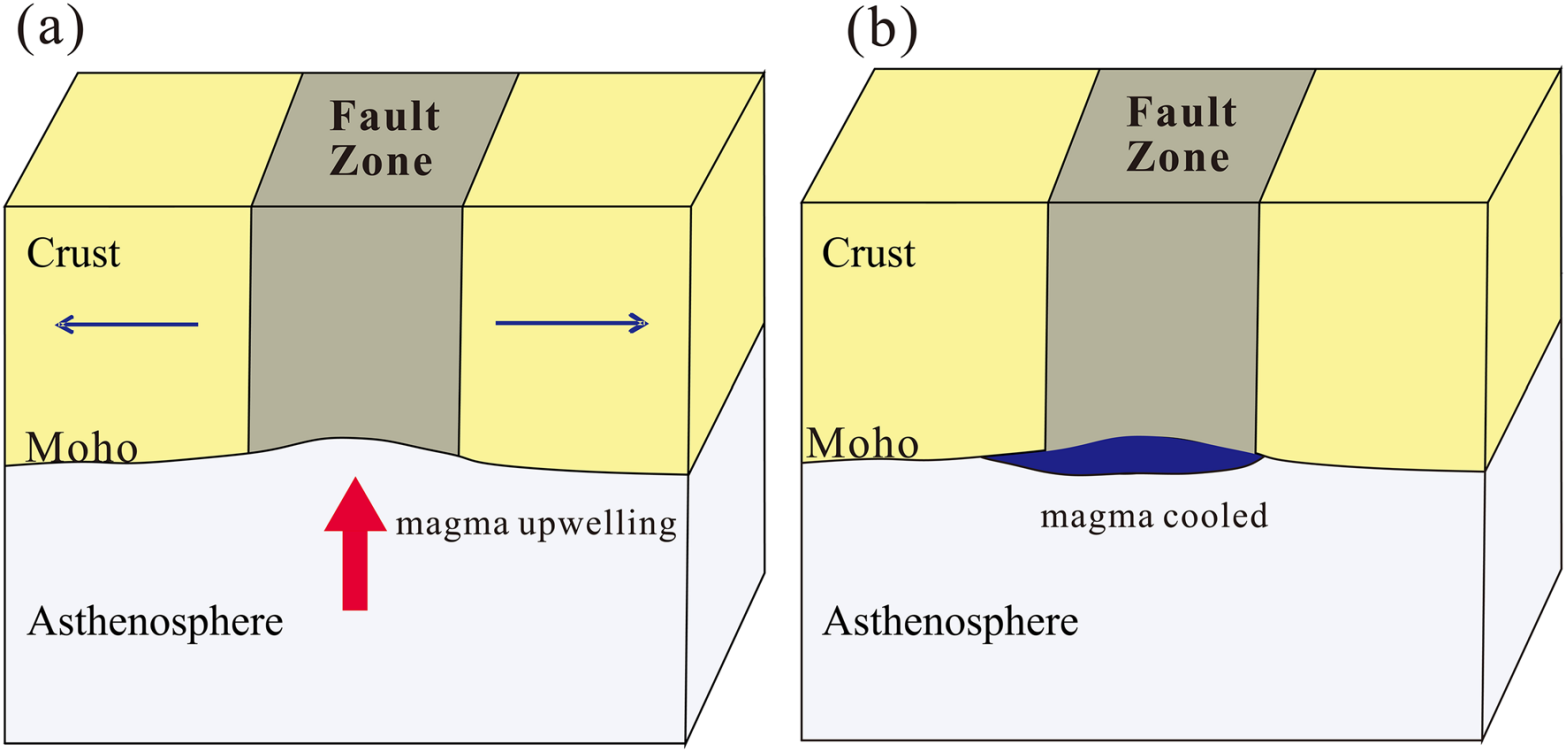
Schematic diagrams illustrating the evolution of the Dunhua-Mishan fault. (**a**) The magma upwelling within the fault zone due to crustal extension in the Late Cretaceous-Paleogene periods. (**b**) The magma upwelling cooled and solidified within the fault zone at the crust-mantle boundary with the development of geological time, contributing to the observed double Moho discontinuities.

The fault-scale crust structure beneath the Dunhua-Mishan fault may be a significant indication of the historical multiple tectonic activities (Fig. 7). It is commonly accepted that the Dunhua-Mishan fault originated in the Early Cretaceous period as a sinistral strike-slip fault zone, caused by the combined effect of the obliquity subduction of the Izenazaki plate with a high speed toward the NNW direction and the final closure of the Mongolo-Okhotsk Ocean in the north of Northeast China^1,11,19,50^. Subsequently, the Dunhua-Mishan fault experienced strong extension activities in the Late Cretaceous-Paleogene periods, which is mainly related to the subduction of the Pacific plate^51,52^. We speculate that the double Moho structure beneath the Dunhua-Mishan fault may be primarily influenced by the strong extension activity, which could caused by the thermal erosion due to asthenospheric upwelling^52–54^. The joint inversion results of ambient noise and receiver functions showed a low-velocity anomaly extending from the lower crust to the upper mantle along the northern segment of the Tanlu fault zone, which could provide additional evidence for the upwelling of mantle material^55^. Geochronological and geochemical analyses based on ^40^Ar/^39^Ar dating suggest the fault zone experienced significant lithospheric thinning and magmas emplacement during the Late Cretaceous-Paleogene periods, which may indicate the time when the double Moho began to develop^44^. Combined with the constraints from previous multiple technologies and our receiver function results, we suggest that the upper Moho could be a remnant feature in the early Cretaceous period, which may represent the ancient crust-mantle boundary. During the latest strong extension activities in the Late Cretaceous-Paleogene periods, the fault suffered magmas emplacement associated with the asthenospheric upwelling. Then the upwelling magma cooled and solidified at the bottom of the lower crust with the development of geological time, contributing to the observed lower Moho discontinuity. The new finding of the double Moho with the fault zone provides critical evidence for the fault evolution and potential fault damage in the future.

## Conclusion

Fault-sacle crustal structure in the Dunhua-Mishan fault is critical for reconstructing the northern segment of the Tanlu fault evolution and geodynamic processes. In this study, we construct fault-scale variations of the crustal structure across the Dunhua Mishan fault based on a temporary linear dense seismic array. Our results reveal the lateral Moho variations at ~ 26–31 km. The slight offset of the Moho discontinuity is observed beneath the south branch of the Dunhua-Mishan fault, probably indicating the Dunhua-Mishan fault is a lithospheric scale fault in the study region. The relatively higher crustal Vp/Vs ratio within the Dunhua-Mishan fault zone suggests may be related to the intrusion of mafic magmatic material. Moreover, the existence of double Moho discontinuities within the fault zone may be influenced by the extensional tectonic activity in the Late Cretaceous-Paleogene periods associated with the retreat of the subducted Pacific plate. More geophysical and geological constraints are required to further evaluate our proposed hypotheses.

## Data and method

The P-wave receiver functions were obtained by deconvolving the vertical component with the radial component with performing the deconvolution algorithm^56,57^. In order to obtain high-resolution of the fault-scale crustal structure of the Dunhua-Mishan fault in the northern segment of the Tanlu fault zone, a linear seismic array was deployed from November 3 to 25, 2019. This array contains 30 temporary seismic stations with an average station spacing of ~ 0.2–0.4 km (Figs. 1 and S1). The station was equipped with a three-component broadband sensor with a sampling rate of 100 points per second. In total, we selected 21 high-quality teleseismic events with magnitude above 5.5 and the epicentral distance ranging from 30° to 95°. Then we removed linear trends and mean values of the raw data and bandpass filtered at 0.4–3 Hz for raw waveforms. Subsequently, we rotated the records from the vertical-north-east (ZNE) system to the vertical-radial-transverse (ZRT) system according to the azimuth distribution. Based on the IASP91 velocity model^58^, we defined a total of 85 s long time windows with 5 s before and 80 s after the arrival time of the direct P-wave, respectively. We established a maximum number of 300 iterations and an iteration error threshold of 0.001. Finally, we used the time-domain iterative deconvolution method to construct the receiver function with a Gaussian width of 5^59^. In total, we obtained 237 high-quality receiver functions.

We constructed a detailed crustal structure along the linear seismic array by using the CCP technique^60^. We obtained the ray-paths of the receiver function by using the IASP91 velocity model^58^. Each amplitude on the receiver function following the direct P-wave was assumed to be generated by the Ps conversion on a seismic discontinuity along the theoretical ray path. After time-to-depth conversion of the receiver function, the amplitude was projected onto the corresponding depth. The stratum beneath the study region was divided into bins with a certain size, and the amplitudes of all the receiver functions in the same bin were summed to enhance conversions amplitudes signals, contributing to the intuitive CCP imaging. In this study, we chose rectangular bins with 2 km lateral width with a sliding distance of 0.35 km along the profile to produce the high-resolution fault-scale crustal imaging. Moreover, we also performed the uncertainty analysis of the CCP stacked amplitudes using bootstrap method with a sample size of 237 receiver functions and 1500 bootstrap iterations (Fig. S3). It can be seen that the uncertainty is small at depths of double positive Ps converted phases.

We calculated the average crustal thickness and Vp/Vs ratio by performing the H-κ stacking algorithm at each station^37^. The H-κ stacking algorithm can determine the optimal crustal thickness and Vp/Vs ratio when Ps, PpPs, and PpSs + PsPs phases of the Moho are stacked coherently. The H-κ stacking uncertainties can be estimated using the bootstrap method with a sample size of all obtained receiver functions at each station and 1000 bootstrap iterations. Here, we chose weighting factors 0.5, 0.3, and 0.2 for Ps, PpPs, and PpSs + PsPs phases of the Moho, respectively. We set Vp to be 6.30 km/s based on the earlier seismological study with an Vp range of 6.25–6.40 km/s^30^. The crustal thickness range and the average Vp/Vs in the crust were respectively chosen within a range of 20–50 km and 1.6–1.9.

### Supplementary Information

Supplementary Figures.

## Data Availability

The seismic waveform data used in the study are archived on a webpage (10.5281/zenodo.10422096).
