# Supplementary material for: Fault-scale crustal structure across the Dunhua-Mishan fault (Tanlu northern segment) constrained from teleseismic P-wave receiver functions
Source: Sci Rep. 2024 Mar 9;14:5823. doi: 10.1038/s41598-024-56620-2 (PMC10924873; doi:10.1038/s41598-024-56620-2)
Supplement: Supplementary file 1 — Supplementary Figures. [file 41598_2024_56620_MOESM1_ESM.pdf]

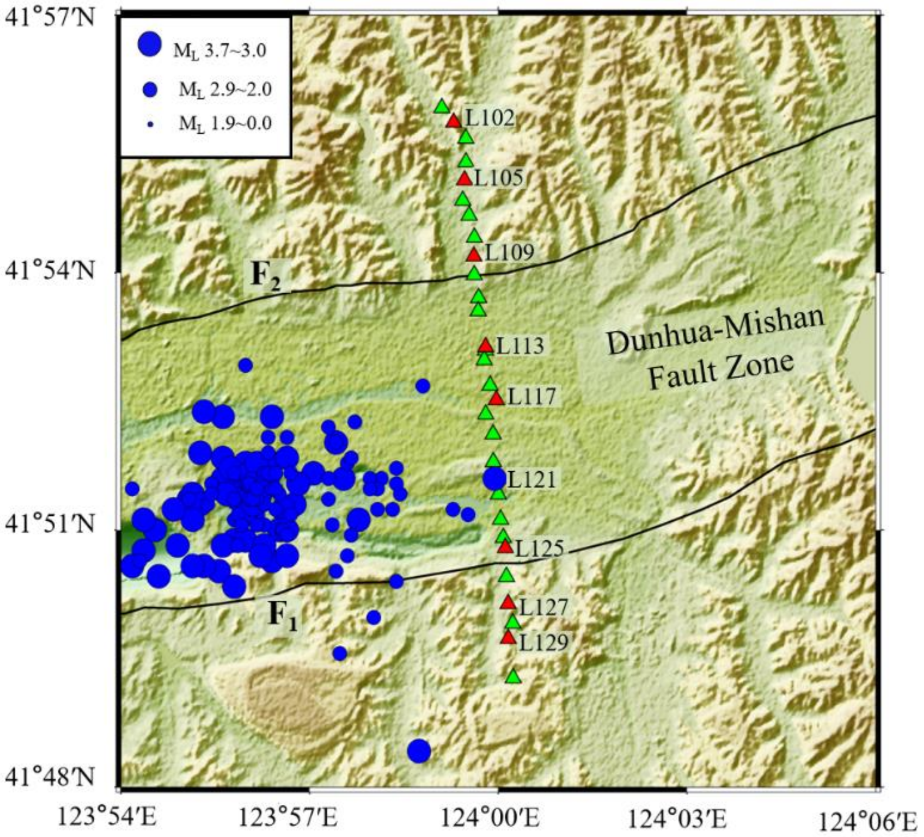

Figure S1. The linear seismic array (green and red triangles) and faults (black lines) in the study region. F1, the south branch of the Dunhua-Mishan fault. F2, the north branch of the Dunhua-Mishan fault. The blue dots represent the seismicity in this region. Imagery is available from the U.S. Geological Survey (https://lpdaac.usgs.gov/products/srtmgl1v003). Figure made with Generic Mapping Tools[60] (GMT v.6.4.0: https://www.generic-mapping-tools.org).

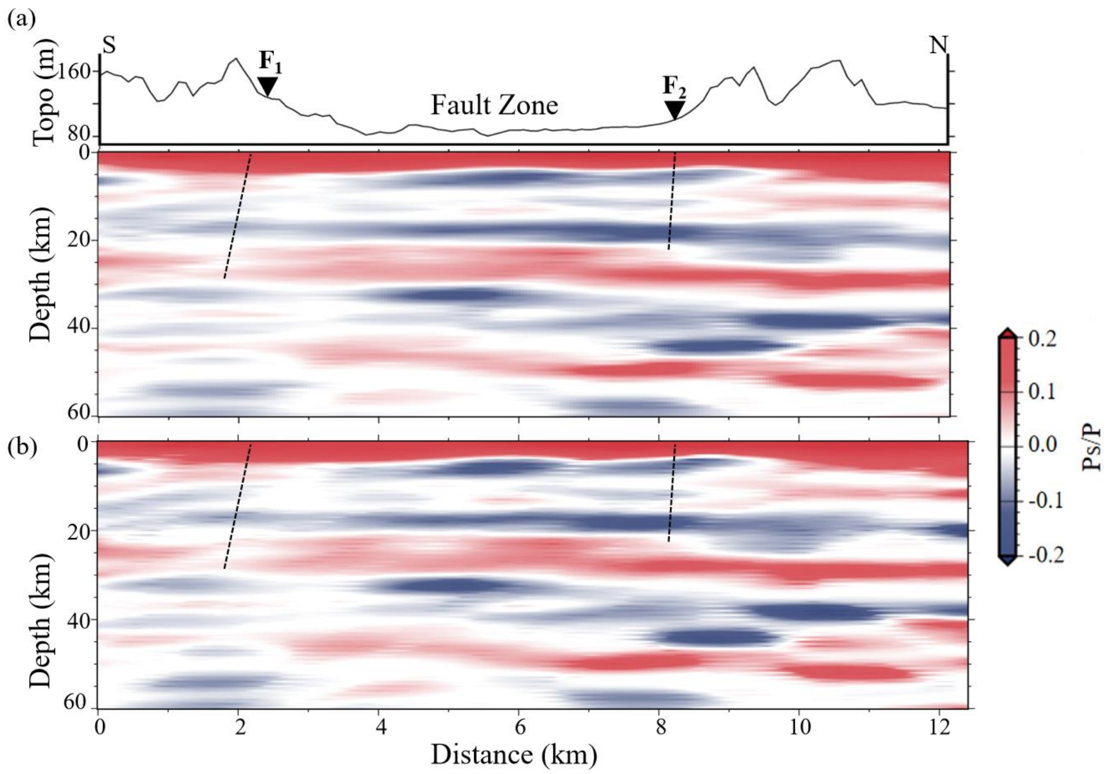

Figure S2. (a) The CCP image of the crustal structure choosing rectangular bins with 2 km lateral width with a sliding distance of 0.35 km along the profile. (b) The CCP image of the crustal structure choosing rectangular bins with 2 km lateral width with a sliding distance of 0.1 km along the profile. Red and blue colors show positive and negative amplitudes, respectively. The top black curve is topography. F_1, the south branch of the Dunhua-Mishan fault. F_2, the north branch of the Dunhua-Mishan fault.

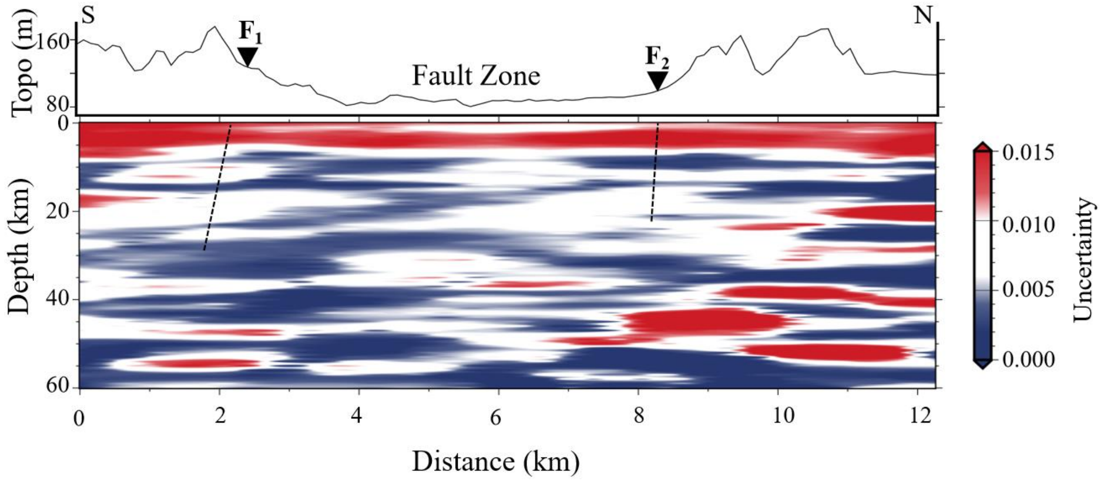

Figure S3. The uncertainty of the CCP stacked receiver function amplitudes using bootstrap method. The top black curve is topography. F1, the south branch of the Dunhua-Mishan fault. F2, the north branch of the Dunhua-Mishan fault.

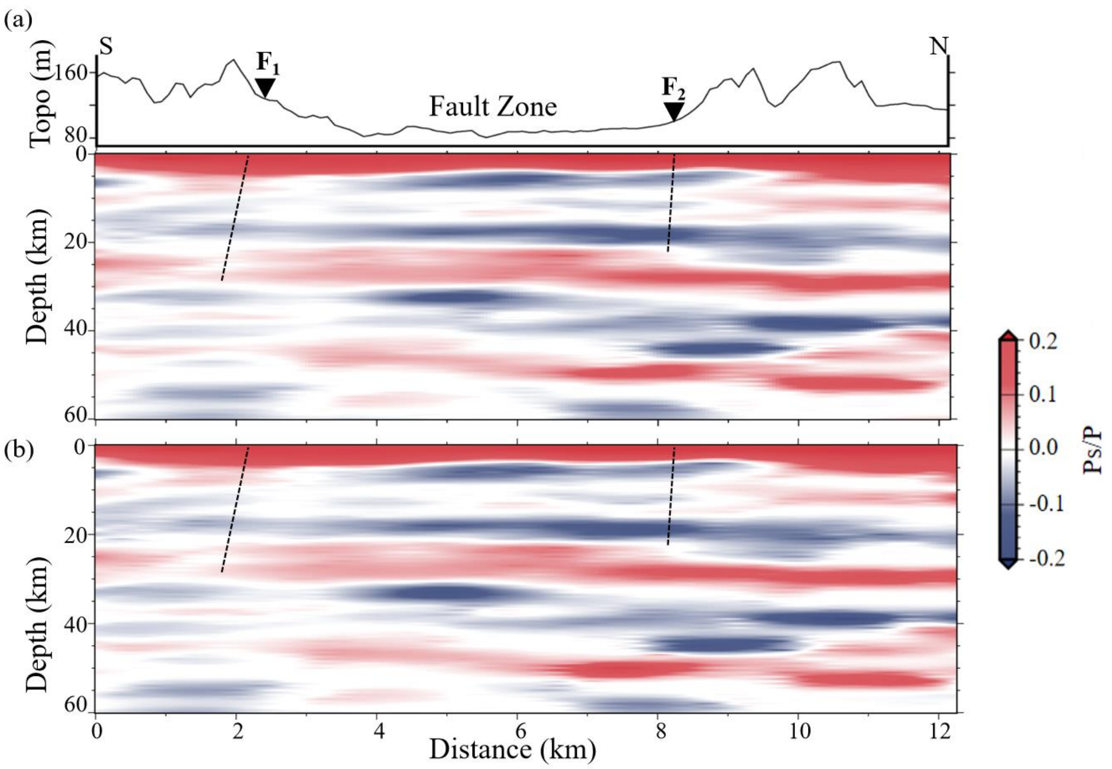

Figure S4. (a) The CCP image of the crustal structure using the IASP91 velocity model in the study region. (b) The CCP image of the crustal structure using the regional 3D velocity model from Shen et al. (2016)[35] in the study region. Red and blue colors show positive and negative amplitudes, respectively. The top black curve is topography. F_1, the south branch of the Dunhua-Mishan fault. F_2, the north branch of the Dunhua-Mishan fault.

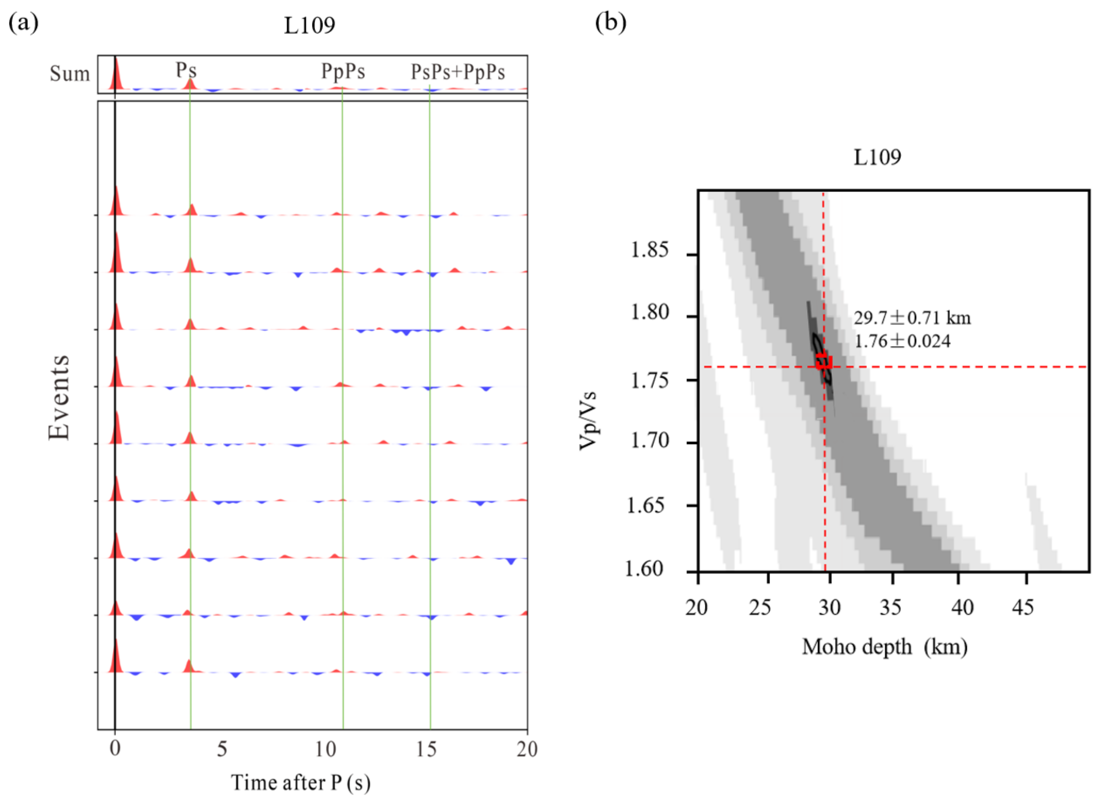

Figure S5. (a) Individual receiver functions at station L109. Green lines represent theoretic arrival times for seismic phases Ps, PpPs, PsPs+PpSs of the receiver functions, respectively. (b) Results of the H-κ stacking at stations L109. The small red square represents the best estimates of crustal thickness and Vp/Vs ratio. The corresponding station codes are shown on the top.

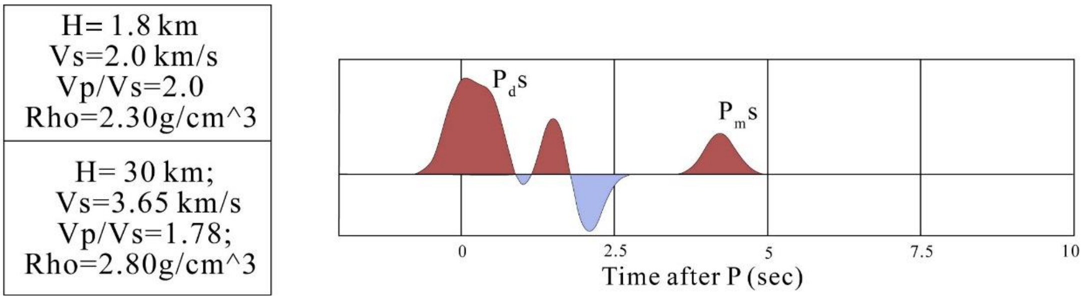

Figure S6. Synthetic Radial RF using the method by Frederiksen and Bostock (2000) to examine the effect of the sedimentary layer on the Moho depth estimation.
